# The Microstructure and Strength of UFG 6060 Alloy after Superplastic Deformation at a Lower Homologous Temperature

**DOI:** 10.3390/ma15196983

**Published:** 2022-10-08

**Authors:** Elena V. Bobruk, Pavel D. Dolzhenko, Maxim Yu. Murashkin, Ruslan Z. Valiev, Nariman A. Enikeev

**Affiliations:** 1 Institute of Physics of Advanced Materials, Department of Physiscs of Metals and Materials Science, Ufa State Aviation Technical University, 450008 Ufa, Russia; 2 Laboratory of Bulk Nanostructured Materials, Belgorod State University, 803015 Belgorod, Russia; 3 Laboratory for Dynamics and Extreme Performance of Advanced Nanostructured Materials, Saint Petersburg State University, 199034 St. Petersburg, Russia; 4 Center for Design of Functional Materials, Bashkir State University, 450076 Ufa, Russia

**Keywords:** superplasticity, aluminum alloys, ultrafine-grained materials, strength

## Abstract

The paper reports on the features of low-temperature superplasticity of the heat-treatable aluminum Al-Mg-Si alloy in the ultrafine-grained state at temperatures below 0.5 times the melting point as well as on its post-deformation microstructure and tensile strength. We show that the refined microstructure is retained after superplastic deformation in the range of deformation temperatures of 120–180 °C and strain rates of 5 × 10^–3^ s^–1^–10^–4^ s^–1^. In the absence of noticeable grain growth, the ultrafine-grained alloy maintains the strength up to 380 MPa after SP deformation, which considerably exceeds the value (250 MPa) for the alloy in the peak-aged coarse-grain state. This finding opens pathways to form high-strength articles of Al-Mg-Si alloys after superplastic forming.

## 1. Introduction

A commercial interest of the automotive and aerospace industries in metallurgical forming under superplastic (SP) conditions is growing nowadays [1,2,3]. During the SP processing, metallic materials are subjected to single operation molding of complex shapes with reduced energy consumption due to the lower resistance of these materials to loading in SP conditions [4]. The single operation SP molding of lightweight materials provides reducing product/vehicle weight and improves the efficiency of processing and the performance of final products. Traditionally, the manufacture of products from light alloys involves sheet or die stamping as a basic forming operation [1,2,3]. The disadvantage of SP deformation is related to a slower rate of the process due to the relatively low strain rates, where superplasticity usually occurs (lower than 10^−4^ s^−1^) accompanied with high temperatures of SP manifestation [5,6]. The implementation of low-temperature and high strain rate SP deformation is, therefore, an important research and development problem of the modern materials science.

To solve this problem, several approached have been developed, which include two main procedures. First, it is necessary to achieve equiaxed homogeneous ultrafine-grained (UFG) microstructures in bulk workpieces, which provide features of low-temperature SP behavior. These microstructures can be formed thanks to specific thermal and deformation treatment using, in particular, severe plastic deformation (SPD) techniques [7]. Second, it is critical to ensure structural stability and maintain a reasonable strength level of the material in the post-SP deformation state.

A special interest of the aerospace and automotive industries is focused on Al alloys to be used as key engineering materials [4]. In particular, Al-Mg-Si alloys (6xxx series) are widely used in both the aerospace (for fuselage shells and other applications) and automotive engineering (for body frame panels and bumpers) due to a well-balanced combination of attractive features, such as strength, formability, weldability, corrosion resistance, low weight, and low cost [1,2,3,8]. Achieving UFG structures in Al various Al alloys allowed for significant improvement of their SP properties. For example, the 6013 alloy demonstrated decreasing the strain rate and temperature of SP after reducing the grain size down to 10 µm. Maximal strain rate sensitivity (*m*) of 0.5 observed at 540 °C for the strain rate in the range of 2 × 10^−4^ to 5 × 10^−4^ s^−1^ with the maximum elongation to failure of 375% was reported in [9,10]. The same material additionally alloyed with Ni, Sc and Zr with a yield strength of 370 MPa showed features of SP at high strain rates. In the temperature range of 440–520 °C and with the strain rates of 1 × 10^−3^ s^−1^ to 1 × 10^−2^ s^−1^, an elongation-to-failure of 350–480% was recorded [11,12].

Extensive studies of UFG Al alloys [13,14,15,16,17] reported on the manifestation of their SP behavior with high values of elongation to failure predominantly at high temperatures (0.6–0.9 *T_m_*, where *T_m_* is the melting point). However, high deformation temperatures lead to degradation of the UFG structure after completing SP process, and hence to a significant decrease in the strength parameters of the final products. This issue limits the potential of Al alloys for industrial application by forming in SP conditions.

The low-temperature SP deformation (approaching 0.5 *T_m_*) of the UFG 6061 Al alloy with a grain size of 400 nm produced by SPD was reported in [18]. At the temperature of 250 °C (0.56 *T_m_*) and the strain rate of 1.7 × 10^−4^ s^−1^, the elongation to failure of 160% was achieved, while the flow stress did not exceed 40 MPa. An increase in the deformation temperature to 540 °C provided elongation of 280% with a flow stress lower than 6 MPa at the strain rate of 3 × 10^−4^ s^−1^. However, the UFG structure notably degraded in the latter case: grain coarsening up to 18 µm occurred. The other researchers reported on the low-temperature SP of the UFG 6063 Al alloy produced by ARB rolling [19]. This alloy exhibited an excellent high strain rate and a low-temperature SP at 300 °C (with the flow peak stress of 40 MPa and maximum elongation of up to 270%) within the nominal strain rates in the range of 5 × 10^−^³ s^−¹^ to 5 × 10^−¹^ s^−¹^. The authors had suggested that the main mechanism responsible for the occurrence of large uniform or SP deformation at 300 °C is associated with dynamic recovery. It was also mentioned that at 350 °C despite lower SP strain rates of 5 × 10^−^² s^−^¹, 5 × 10^−^³ s^−^¹ and high elongations, the microstructural instability was observed [19], which is undesired for industrial applications.

In the meantime, the recent works have shown that the formation of UFG states in Al alloys with a regulated distribution of alloying elements to form secondary hardening phases as well as segregations or interlayers along grain boundaries can lead to a unique combination of properties, when the strength, plasticity and electrical conductivity of the material simultaneously increase [20,21,22]. In addition, it was shown that grain refinement to the nanoscale range allows for realizing the SP effect at low and even ultralow (below 0.5 *T_m_*) temperatures [23,24,25,26,27,28,29]. Under such conditions, the thermally induced degradation of the UFG microstructure could be prevented, which would ensure maintaining high strength of Al alloys after SP forming. Therefore, it is important to study microstructural stability and mechanical performance of the UFG Al alloys in the post-deformation state after low/ultralow-temperature SP. Maintaining superior mechanical behavior after SP deformation will open up new possibilities for the development of promising lightweight and high-strength complex products shaped via SP forming.

Thus, the purpose of this work is to study the features of the UFG structure evolution after ultralow-temperature SP and the possibility of maintaining high post-deformation mechanical strength in an Al-Mg-Si alloy. For the first time, we show the possibility of achieving a high-strength state of the 6060 Al alloy after SP forming at ultralow temperature (*T* < 0.5 *T_m_*) by retaining the UFG structure, which was hardly possible at SP at higher temperatures reported by the other studies summarized in the state of art. We also report on the thorough study of the post-deformation microstructure and texture evaluation in the SP deformed area of tensile tested samples in different ranges of strain rates and temperatures.

## 2. Materials and Methods

The object of this study is an Al-Mg-Si alloy (AA6060). The chemical composition of the alloy (Table 1) has been qualitatively analyzed by energy dispersive spectroscopy on a JSM-6490LV scanning electron microscope (JEOL Ltd., Tokyo, Japan) equipped with an INCA X-Act attachment (Oxford Instruments plc, Tubney Woods, Abingdon, UK).

As-received specimens of the studied alloy were produced by conventional hot-pressing and had a typical coarse-grained structure of semi-finished products after pressing. The width of the fibers was approximately 20 µm and the length was 250 µm. Following [30] prior to SPD processing, the as-received specimens were solid-solution treated by annealing at a temperature of 530 ± 5 °C (which is below the melting temperature of Mg, Al and the other components of the alloy [31]) for 1 h followed by quenching with water to assist maximum grain refinement during SPD and to reserve a potential for additional hardening by controlled precipitation hardening. The homogenized specimens were processed by SPD no later than 1.5 h after the heat treatment to utilize the maximum effect of quenching [30].

The samples of the required geometry were prepared by a high-precision two-coordinate wire-cutting spark erosion machine ARTA-120 (OOO “NPK Delta-Test”, Fryazino, Russia) in water at room temperature to eliminate the thermal effects. To nanostructure the material, the high pressure torsion (HPT) technique [7] was applied to the disks of 20 mm in diameter and 1.4 mm in thickness at room temperature under the pressure of 6 GPa and HPT rotation rate of 0.2 rpm. The number of revolutions of the rotating anvil (*n*) during HPT was 20. The structure and mechanical properties of the HPT processed alloy were precisely studied on the samples representing a region located at the half radius of an HPT disk.

**Figure 1:**
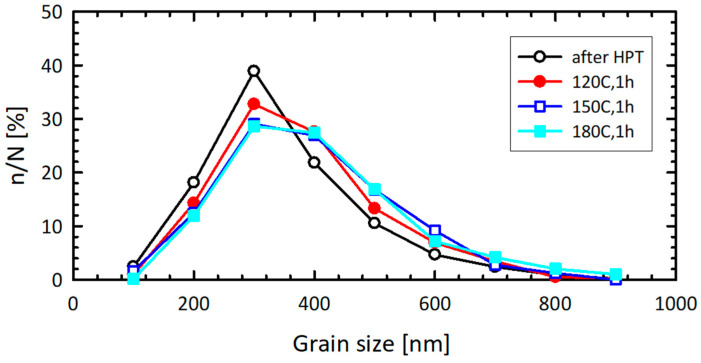
Grain size distributions of the UFG 6060 alloy after annealing at 120 °C, 150 °C and 180 °C for an hour.

Mechanical tests were carried out at the temperatures of 120 °C, 150 °C and 180 °C. The thermal stability of the UFG 6060 alloy microstructure at these temperatures was examined by one-hour annealing (Figure 1). The grain size and grain size distribution proved to be stable over the entire range of the testing temperatures.

Tensile tests were carried out with the help of a universal INSTRON 5982 testing system with Bluehill 3 software and a computer system for evaluating mechanical characteristics (ToolWorks Inc., Norwood, MA, USA). Tensile tests were performed at the strain rates of 10^−2^, 5 × 10^−3^, 10^−3^, 5 × 10^−4^ and 10^−4^ s^−1^. The gauge of the tensile test samples was 3.3 × 1.1 × 0.6 mm^3^. Strain rate sensitivity coefficient values were calculated according to the formula:

$$m=\frac{\partial \ln\left(\sigma \right)}{\partial \ln\left(έ\right)},$$

where σ is the flow stress, έ is the true strain rate.

In order to evaluate the room temperature mechanical performance of the UFG alloy in the structural state formed after SP deformation, a specific procedure was used for the state exhibiting the best SP performance (tensile deformed at 150 °C). At the first stage, the sample was tested under SP conditions at 150 °C and the strain rate of 5 × 10^−3^ s^−1^ until it was elongated up to approximately 50%. After such deformation, the sample was cooled in water at room temperature. At the second stage, after control measurements of the gauge, the samples were tensile tested at room temperature with the strain rate of 5 × 10^−4^ s^−1^. Vickers hardness (HV) was measured on a Micromet-5101 device (Buehler Ltd., Lake Bluff, IL, USA) with a diamond pyramid indenter, a load of 1 N and a dwell time of 15 s. Measurements were taken with the increments of 0.3–1.0 mm, with the total number of measurements of at least 10. The hardness values were determined automatically using the OmniMet MHTFS software.

The microstructure was studied using a JEOL JEM-2100 (JEOL Ltd, Tokyo, Japan) transmission electron microscope (TEM) operated at 200 kV. For the TEM analysis, the disc-shaped samples of 3 mm in diameter and 0.1–0.15 mm in thickness were twin jet electropolished at a temperature of −25–30 °C and a voltage of 10–12 V. Jet polishing of TEM foils was carried out on a Tenupol-5 automatic electrolytic thinning unit in a solution of 20% nitric acid (HNO_3_) GOST 4461 and 80% methanol grade B (CH_3_OH) GOST 2222.

Microstructure parameters were analyzed using the electron backscatter diffraction technique (EBSD). To produce a suitable surface, each specimen was mounted and mechanically polished using conventional metallographic procedures followed by a final electro-polishing with a suspension of colloidal silica. EBSD was then conducted by FEI Quanta 600 (FEI Company, Hillsboro, OR, USA) field-emission-gun scanning electron microscope equipped with TSL OIM^TM^ software. EBSD orientation maps were collected from the transverse direction of the specimens subjected to HPT or tensile specimens. Orientation maps were acquired for each material condition using a scan step size of 0.05 μm. To improve the reliability of the acquired EBSD data, small grains comprising 5 or fewer pixels were automatically removed during the grain reconstruction procedure by the MTEX software used for the EBSD analysis [32]. Due to the limited angular accuracy of EBSD, boundaries with misorientations below 2° were excluded from consideration. Last, a 15° threshold was applied to differentiate low-angle boundaries (LAGBs) and high-angle grain boundaries (HAGBs).

## 3. Results

### 3.1. Microstructure and Mechanical Properties of 6060 Alloy after HPT

**Figure 2:**
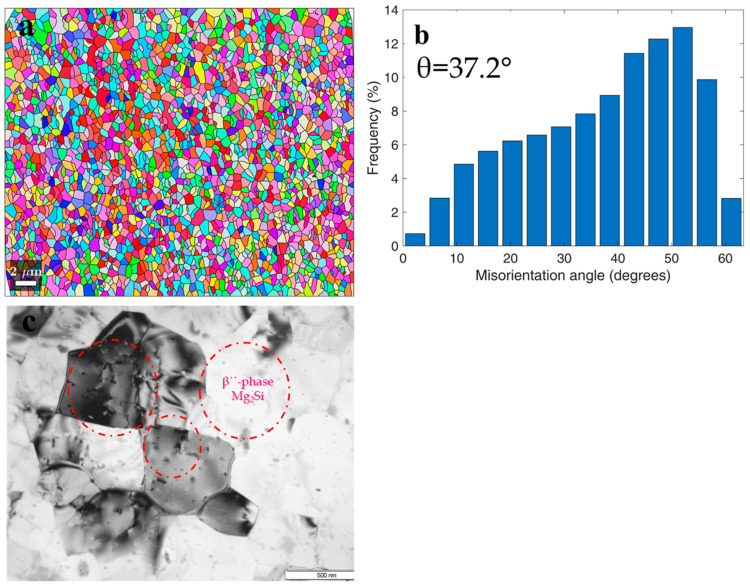
The microstructure of the UFG 6060 alloy produced by HPT (**a**) orientation map (transverse direction); (**b**) grain boundary misorientation distribution; (**c**) bright field TEM image revealing nanoscale precipitates and dislocations.

HPT processing of the 6060 alloy samples resulted in the development of a homogenous UFG structure predominantly formed by HAGBs as demonstrated by EBSD microstructure analysis (Figure 2a,b) and consistent with earlier studies [29]. The average size of grains with close to equiaxial shape was 0.95 ± 0.05 µm, the shape factor (*Kf*) was 1.2, the volume fraction of HAGBs was 91%, the maximum misorientation angle of the grain boundaries was in the range of 50–60°, the average misorientation angle was 37.2°, and the average HAGBs misorientation angle was 38.7°.

TEM analysis revealed separate nanosized precipitates (with the size of less than 2 nm) in the interiors and at the boundaries of ultrafine grains (Figure 2c). Bundles of lattice dislocations were also resolved inside individual grains. Following the results of earlier studies, these precipitates were identified as β″-Mg_2_Si phase (a metastable modification of the Mg_2_Si-phase) [33]. The formation of similar UFG microstructure with nanosized Mg_2_Si particles resulted from dynamic precipitation was described in more detail in [33,34], where it was also noted that, in addition to precipitation, the segregation of alloying elements along grain boundaries occurred.

UFG microstructure allowed for increasing the yield stress (σ_0.2_) of the alloy to 500 ± 4 MPa and the ultimate tensile strength (σ_UTS_) to 530 ± 4 MPa accompanied with elongation to failure of 6.5 ± 0.2%. The strength of the UFG 6060 alloy by two times exceeds values typical for the coarse-grained alloy after conventional heat treatment (T6) to achieve the maximum strength (Table 2).

### 3.2. Mechanical Properties of UFG 6060 Alloy at Elevated Temperatures

**Figure 3:**
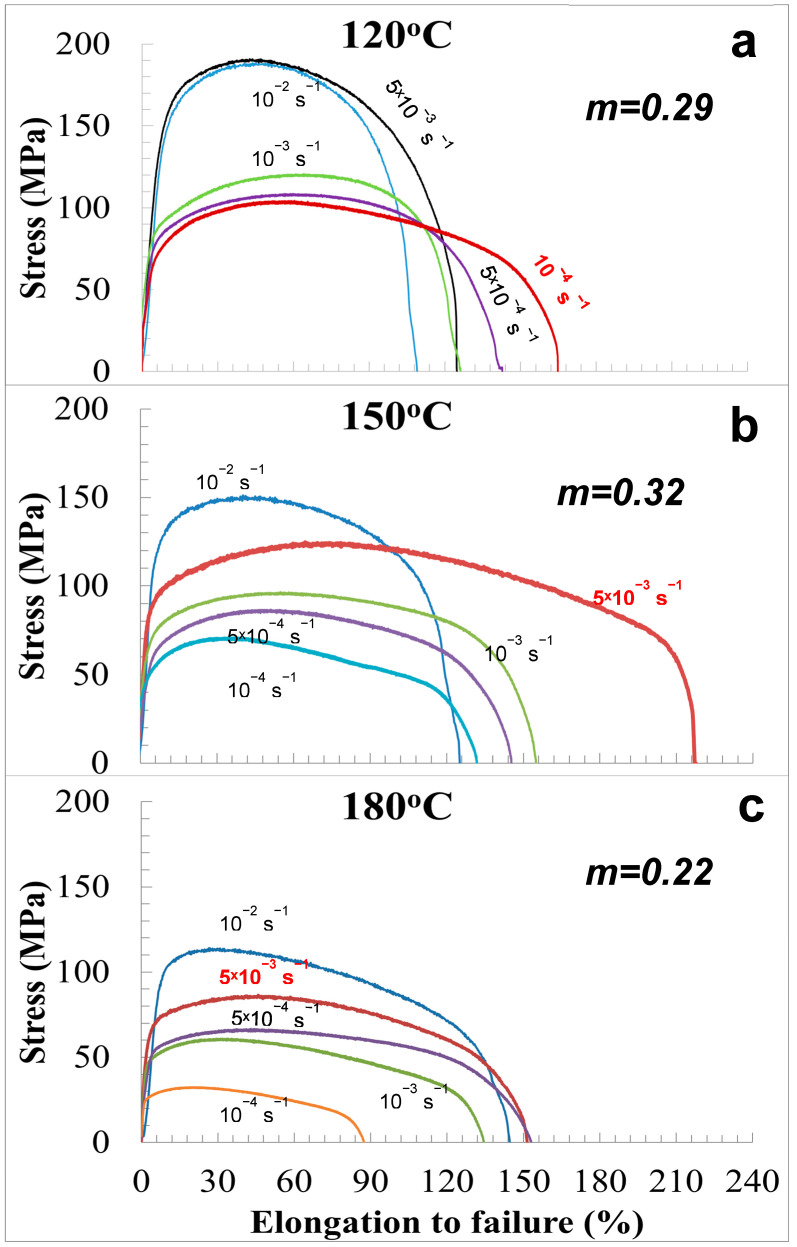
The stress−strain curves for the UFG 6060 alloy for different strain rates and deformation temperatures of: (**a**) 120 °C (0.42 *T_m_*); (**b**) 150 °C (0.46 *T_m_*); and (**c**) 180 °C (0.48 *T_m_*).

Mechanical tensile tests to reveal SP features during deformation of the UFG 6060 Al alloy were performed at temperatures of 120–180 °C in the strain rate range of 10^−2^ to 10^−4^ s^−1^ in accordance with [35]. Figure 3 shows typical engineering “stress (σ)-strain (ε)” curves, obtained at deformation temperatures of 120 °C (0.42 *T_m_*), 150 °C (0.45 *T_m_*), and 180 °C (0.47 *T_m_*). The figure indicates that the flow stresses decrease with increasing strain rate and temperature.

For all cases, the curves are characterized by pronounced strain hardening and a smooth decrease in the flow stress after achieving peak values. As it is assumed, the observed strain hardening is related to multiple intragranular slip, with no steady-state flow stage as observed in [36,37]. As a result of the tests, the maximum elongations reached values of 150–220% at strain rates of 5 × 10^−3^ s^−1^–10^−4^ s^−1^, and the *m* parameter was within 0.29–0.32. Deformation curves at 180 °C did not demonstrate considerable scattering in elongation to failure with variation in the strain rate.

Considering the values of calculated *m* and achieved elongations to failure, it can be stated that the UFG 6060 alloy demonstrates the features of SP behavior at ultralow temperatures (*T* < 0.5 *T_m_*) [15,16]. For a further detailed analysis, we chose three states of UFG 6060 alloy after SP deformation with the modes at which the maximum elongations and *m* parameters were observed at every testing temperature.

### 3.3. Influence of SP Deformation on the Microstructure of UFG 6060 Alloy

In order to reveal the effect of low-temperature SP deformation on the microstructure of UFG 6060 alloy, the EBSD and TEM studies were conducted in the SP-deformed gauge areas of the fractured UFG samples after tensile tests at different temperatures.

**Figure 4:**
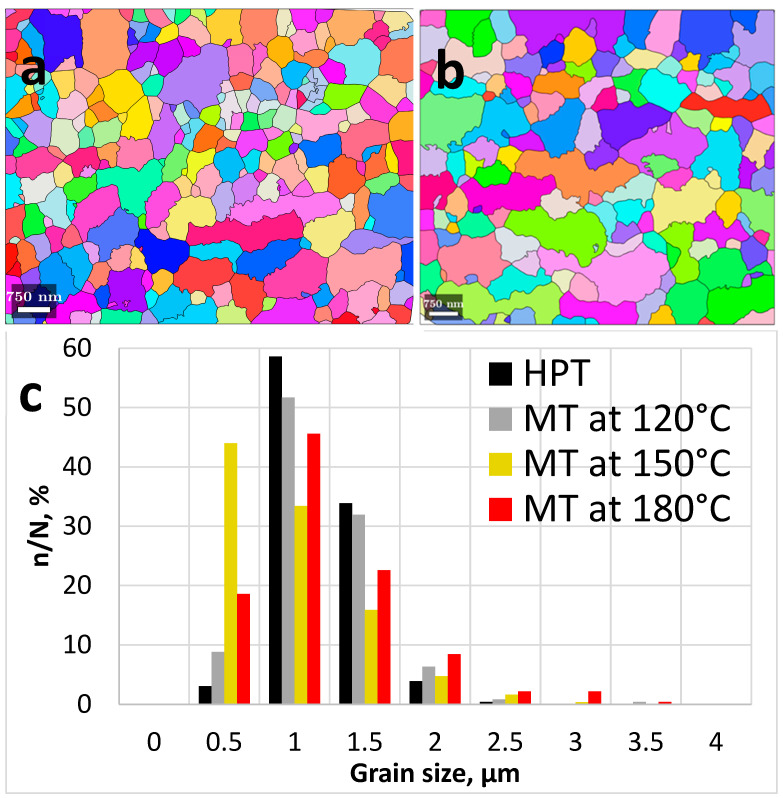
Orientation maps for the gauge areas of the fractured UFG tensile samples after deformation at (**a**) 150 °C and (**b**) 180 °C. (**c**) Grain size distributions for the 6060 alloy after HPT and after HPT and mechanically testing (MT) at 120 °C, 150°C and 180 °C.

Figure 4 indicates that the grain size and shape in the UFG alloy slightly changed after tensile deformation. Note that Figure 4a,b is plotted to show the grains shaped by HAGBs (with misorientations >15°) to demonstrate significantly refined grain structure in both states. According to [35,36], a high fraction of HAGBs in the as-produced UFG 6060 alloy prevents abnormal grain growth.

**Figure 5:**
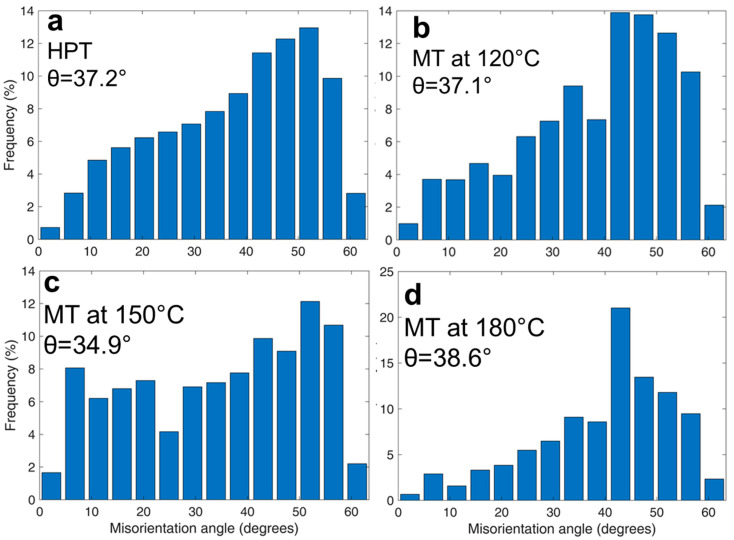
Distribution diagram of boundary misorientation angles depending on their volume fraction of UFG 6060 alloy after (**a**) HPT; mechanical testing at (**b**) 120 °C; (**c**) 150 °C (**d**) and 180 °C.

The EBSD analysis of the alloy after SP deformation at 120 °C showed that the fraction of LAGBs and HAGBs did not change, amounting to 9% and 91%, respectively (Figure 5b). The average misorientation angle was 37.1°, the average HAGB misorientation angle was 38.8°, the values of grain boundary misorientation angles shifted closer to the range of 40–50°, and the average grain size was 0.90 ± 0.05 µm, *K_f_* = 1.3.

After SP deformation at 150 °C, the fraction of LAGBs increased to 16% and HAGB fraction decreased to 84% (Figure 5c). The average size of misorientation angle was 34.9°, the average HAGBs misorientation angle was 34.5°, the maximum values of grain boundary misorientation angle were in the range of 50–60°, and the average grain size was 0.8 ± 0.05 µm, *K_f_* = 1.5 (Figure 5c).

SP deformation at 180 °C led to decreasing of the LAGBs fraction to 6% and increasing HAGBs fraction to 94% (Figure 5d). The average misorientation angle was 38.6°, the average HAGBs misorientation angle was 40.8°, the peak of grain boundary misorientation angle was observed at 45°, and the grain size was 0.95 ± 0.05 µm, *K_f_* = 1.5 (Figure 5d).

**Figure 6:**
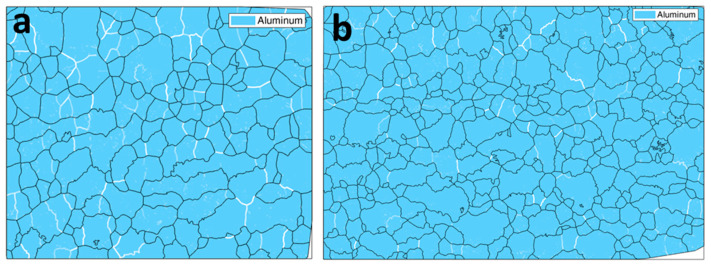
Grain boundary reconstruction by EBSD data showing appearance of LAGBs (white lines with intensities proportional to misorientation) in the microstructure of the gauge section for the UFG 6060 alloy specimen tensile tested with the strain rate of 5 × 10^−3^ s^−1^ at (**a**) 150 °C; (**b**) 180 °C.

Although the microstructures look equiaxial, the quantitative EBSD analysis revealed the presence of elongated grains subdivided by LAGBs. Typical examples are shown in Figure 6 where LAGBs with misorientations less than 15° are colored in white. Figure 6 indicates that low angle subgrain boundaries split larger elongated grains. Figure 6a demonstrates visibly more pronounced formation of low-angle substructures in the specimen tested at 150 °C than at 180 °C (Figure 6b) and this is quantitatively confirmed by Figure 5c as compared to the other distributions in Figure 5. These observations suggest that the appearing equiaxial microstructure may have evolved by splitting of elongated grains, which assumes that extensive intragranular slip has occurred. This is consistent with the data published recently in [37,38,39,40]. It also well agrees with the recent findings reported in [41] which revealed that the formation of pronounced substructure within elongated grains can be a dominant mechanism of superplastic flow. Intensive formation of LAGBs as indicated by Figure 6 as well as by Figure 5c as compared to Figure 5a,b,d is thus consistent with the pronounced manifestation of SP by UFG 6060 Al alloy during tensile testing at 150 °C.

**Figure 7:**
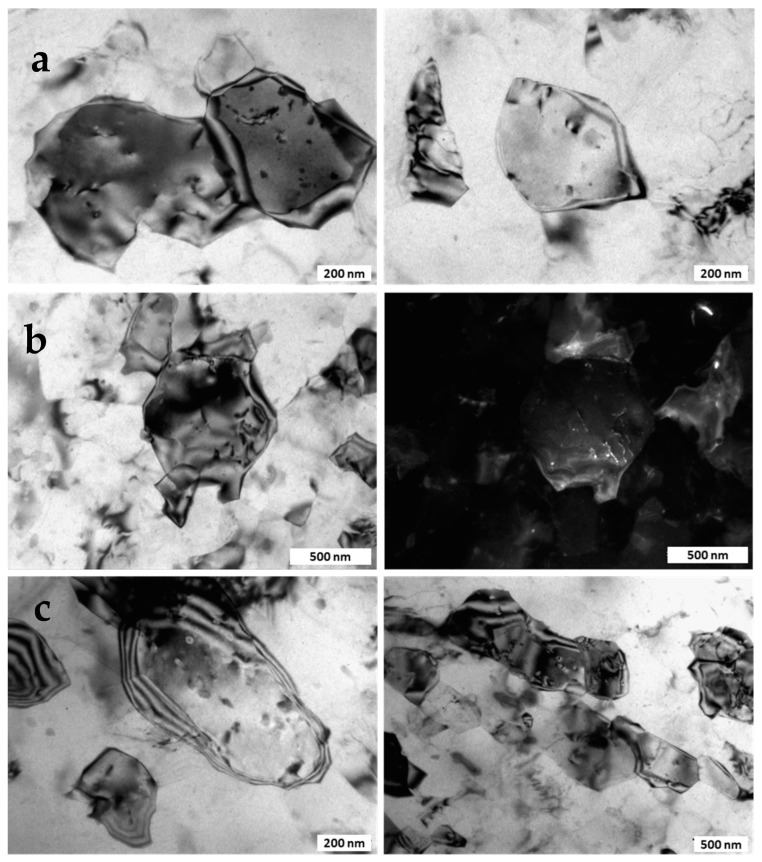
TEM bright field images of the UFG structure revealing precipitates and dislocations after SP deformation at: (**a**) 120 °C; (**b**) 150 °C and (**c**) 180 °C.

**Figure 8:**
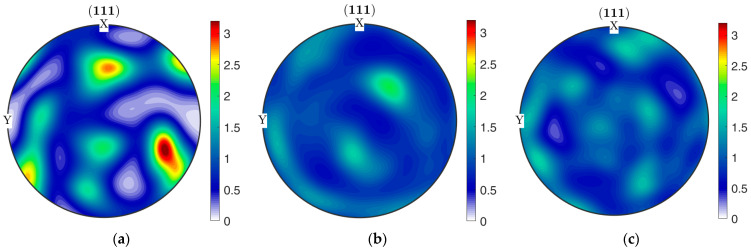
(111) pole figures for the UFG 6060 alloy produced by (**a**) HPT and after subsequent SP deformation at (**b**) 150 °C and (**c**)180 °C.

TEM analysis revealed accompanying decomposition of the solid solution during SP deformation; the dynamic precipitation was more pronounced at the deformation temperature of 180 °C. The size of globular secondary Mg_2_Si precipitates was 10–15 nm (Figure 7). The density of lattice dislocations became higher with an increasing deformation temperature.

(111) pole figures calculated from by EBSD orientation data represent local texture formed in the UFG 6060 alloy after HPT and subsequent SP deformation at 150°C and 180 °C (Figure 8). For illustrative purposes, pole figures for HPT state were rotated to be aligned with the shear direction. The pole figure for the UFG alloy produced by HPT (Figure 8a) demonstrates features of deformation texture with moderate intensity of maximal pole density (the texture index denoting the deviation from random texture consisted 2.47). SP deformation at both temperatures led to the notable texture scattering visible in pole figures (Figure 8b,c), the texture index decreased to 1.22 and 1.31, respectively. Thus, SP deformation results in reorientation of grains toward random texture. This process can be induced by intensified non-crystallographic deformation modes, such as grain boundary sliding or rotation.

### 3.4. Mechanical Properties UFG 6060 Alloy after SP Deformation

The microhardness measured after SP deformation are consistent with the microstructure studies. Higher microhardness of the material was evaluated after tensile tests: at 120 °C-108 ± 5 Hv, while at 150 °C it was 107 ± 5 Hv and at 180 °C it was 85 ± 4 Hv. The hardness after the SP at 180 °C decreases to the level typical for the coarse-grained alloy subjected to T6 treatment (Table 2).

**Figure 9:**
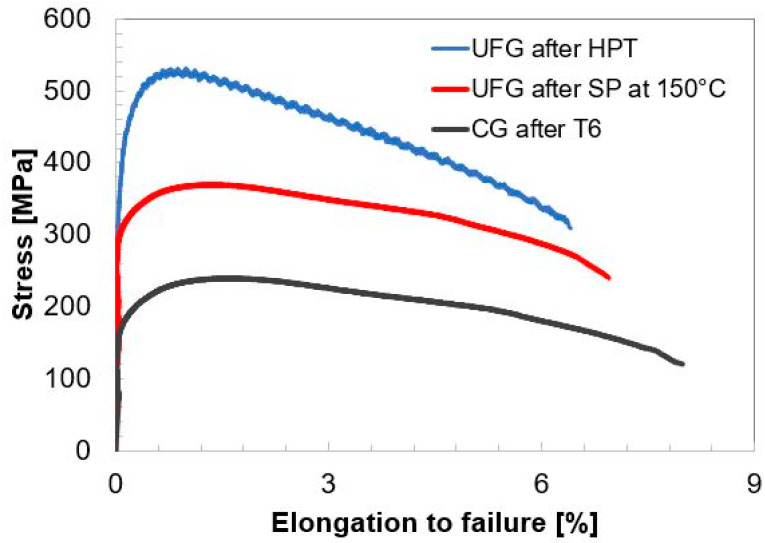
The room temperature tensile curves for the T6-treated coarse-grained and for the UFG 6060 alloy in the states after HPT and after SP at 150 °C.

To assess the strength of UFG Al alloy 6060 after SP deformation, tensile tests were carried out at room temperature on samples subjected to 50% of SP deformation at 150 °C with a strain rate of 5 × 10^−3^ s^−1^. This post-SP deformation state was selected as exhibiting the most pronounced SP behavior with the maximum elongation to failure (220%) and *m* = 0.32. The results of mechanical tests at room temperature are shown in Figure 9 for the UFG 6060 alloy in the as-produced state and in the state after SP deformation. Stress–strain curves for the 6060 alloy in the coarse-grained state after T6 heat treatment are also presented for comparison. Figure 9 and Table 2 indicate that UFG 6060 alloy maintains high strength of 380 ± 3 MPa and reasonable ductility of 7 ± 0.3% after ultralow-temperature SP deformation.

## 4. Discussion

As shown in [33,34], the formation of the UFG structure in the 6060 Al alloy in combination with nanoscale precipitates and segregation of alloying elements in the interiors and boundaries of grains allowed for achieving high strength of 530 MPa. For the first time we show that UFG 6060 alloy can demonstrate SP behavior at ultralow temperatures (below 0.5 *T_m_*). Observations suggested that the equiaxial microstructure may have evolved by splitting of elongated grains, which assumes that extensive intragranular sliding has occurred, this observation is consistent with the data reported recently in literature [37,38,39,40]. According to [14,42], fine grains facilitate grain boundary sliding and dislocation accommodation; therefore, superplastic behavior can be observed at higher strain rates and lower temperatures. The maximum value of elongation was obtained at a temperature of 150 °C, at a strain rate of 5 × 10^−4^ s^−1^. The uniform distribution of nanosized metastable Mg_2_Si particles (2–10 nm in size), as well as grain boundary segregation [14,42], may have an important role in suppressing dynamic grain growth and ensuring stable flow during SP deformation at this temperature. An increase in the deformation temperature to 180 °C leads to an increase in the average size of the secondary hardening phase to 15 ± 0.5 nm, which suggests certain grain coarsening, a decrease in elongation to 160%, and a reduction of the *m* parameter to 0.29, alongside the stability of the grain structure during low-temperature SP deformation being maintained by a high fraction of HAGB (more than 90%) in the UFG state produced by HPT. It can be deduced that the stability of the UFG 6060 alloy under SP conditions is one of the key factors contributing to the observed SP features.

## 5. Conclusions

Ultralow-temperature (T < 0.5 *T_m_*) superplastic behavior is reported for the UFG 6060 Al alloy demonstrated by tensile tests carried out at 120 °C (0.42 *T_m_*), 150 °C (0.45 *T_m_*) and 180 °C (0.47 *T_m_*) at different strain rates. The microstructure parameters of the UFG 6060 alloy such as ultrafine grain size and a large portion of high-angle boundaries do not drastically change after SP deformation, however, the enhanced formation of low angle boundaries is observed under conditions where the most prominent superplastic behavior occurs. Grain boundary sliding and intragranular slip with subgrain boundary formation appear to notably contribute to the superplastic flow. We show that the strength of the UFG 6060 alloy after SP deformation at the temperature of 150 °C (0.45 *T_m_*) and the strain rate of 5 × 10^−4^ s^−1^ can be as high as 380 MPa in combination with elongation to a failure of 7%. Such strength parameters notably exceed those typical for the standard T6-treated 6060 alloy. This finding opens pathways to develop high-strength products of Al-Mg-Si alloys by forming in superplastic conditions.

## Figures and Tables

**Table 1:** Chemical composition of the studied alloy (wt%).

Mg	Si	Cr	Fe	Zn	Ti	Mn	Al
0.60	0.60	0.50	0.20	0.15	0.10	0.10	balance

**Table 2:** Mechanical properties of 6060 Al alloy in different states.

Treatment/ Structural State	Ϭ_0.2_, MPa	Ϭ_UTS_, MPa	δ, %	Hv
Quenching/coarse grained	120 ± 2	185 ± 3	20.0 ± 0.5	40.0 ± 2
T6/coarse grained	180 ± 2	240 ± 2	8.0 ± 0.4	80.0 ± 2
HPT at RT/UFG HPT at RT+MT at 150°C/UFG	500 ± 4 **360** **±** **3**	530 ± 4 **380 ± 3**	6.5 ± 0.2 **7.0** **±** **0.3**	118.0 ± 5 **107.5 ± 5**

## Data Availability

The raw and processed data required to reproduce these results are available by reasonable request.
